# Antibiotic resistance pattern of *Staphylococcus aureus* with reference to MRSA isolates from pediatric patients

**DOI:** 10.2144/fsoa-2019-0122

**Published:** 2020-02-24

**Authors:** Raja Ram Gurung, Prashanna Maharjan, Ganga Gharti Chhetri

**Affiliations:** 1 Tri-Chandra Multiple Campus affiliated to Tribhuvan University, Department of Microbiology, Kirtipur, Kathmandu 44600, Nepal; 2 Biotechnology Society of Nepal (BSN), Rani Devi Marg, Kathmandu 44600, Nepal; 3 Amrit Science Campus affiliated to Tribhuvan University, Department of Microbiology, Thamel, Kathmandu 44600, Nepal

**Keywords:** antibiotic resistance, inducible clindamycin-resistant (ICR) test or D-zone test, macrolide-lincosamide-streptogramin B (MLS_B_) phenotype, methicillin-resistant *Staphylococcus aureus* (MRSA), Nepal

## Abstract

**Aim:**

The extent of methicillin-resistant *Staphylococcus aureus* (MRSA) infection in Nepalese children is largely unknown.

**Materials & methods:**

Six hundred and seventy-two clinical samples collected from 232 patients between June and November 2016 were processed in a microbiology laboratory.

**Results:**

Out of 300 culture-positive samples, 52 (17.3%) were *S. aureus* isolates. Among those 52, 39 (75.0%) were found to be MRSA. The infection rate of *S. aureus* was shown to be higher in inpatients (55.7%) compared with outpatients (44.3%) at p = 0.637, 95% CI. Thirteen types of antibiotics were used in the antibiotic susceptibility test. MRSA isolates showed 100 and 0% resistance to penicillin and vancomycin, respectively. The D-test showed inducible clindamycin-resistant phenotype in 15.4% of MRSA isolates.

**Conclusion:**

This demonstrates the utmost need for routine testing for MRSA in Nepalese hospitals.

## Lay abstract

The problem of antibiotic resistance is rising worldwide. Methicillin antibiotic-resistant *Staphylococcus aureus* (i.e., MRSA) infection is one of the bacterial infections that contribute to increased health and economic burdens. An accurate understanding of the MRSA infection status in Nepalese children is not available to date. Thus, the current research was performed in a pediatric hospital. We also discovered that MRSA is not tested routinely and thus we recommend it should be monitored regularly, as with other bacterial infections.

*S. aureus* remains ubiquitous in the environment and normal flora of animals. It is a commensal as well as pathogenic bacterium [1]. It is known to occur as normal flora in the skin of an estimated 20% of the world population without causing any harmful effects and is persistently carried. Moreover, 60% of the population carries it occasionally during their life time [2,3]. However, it is considered to be an opportunistic pathogen for humans and animals if it gets an opportunity to enter the bloodstream and tissue [1,2]. Generally, it becomes infectious only when it is able to enter into the skin or mucous membrane punctured by a penetrating objects [4]. It is found to cause a range of illness from minor skin infections such as pimples, impetigo, boils, cellulitis, scalded skin syndrome, folliculitis, furuncles, carbuncles and abscesses, to life-threatening diseases such as pneumonia, osteomyelitis, meningitis, Toxic Shock Syndrome, endocarditis and septicemia [5].

Methicillin-resistant *Staphylococcus aureus* (MRSA) is a strain of *S. aureus* that has acquired resistance to β-lactam antibiotics, which include penicillins and cephalosporins. MRSA strains are versatile and significant nosocomial pathogens, often causing postsurgical wound infections almost exclusively of hospital origin, as described in 1961 [6]. MRSA infections account for 20–80% of all nosocomial *S. aureus* infections in many centers across the world [7,8] and lead to increased mortality, morbidity hospital stay and costs [9,10]. WHO has reported that 64% of MRSA-infected patients are more likely die than non-MRSA-infected patients [11]. MRSA may transmit from person to person by physical contact and rarely by air. The nasopharynx is the main ecological niche of the *S. aureus* [12], although it is found in almost all body parts. With little change in overall mortality, the frequency of community-acquired MRSA and hospital-acquired MRSA infections have increased steadily. They contribute to the failure of empirical therapy. Treatment of these infections has become more difficult because of the emergence of multidrug resistance (MDR) [13]. MDR is defined as resistance to three or more antimicrobial classes. MRSA raises greater concern because of its high virulence capacity [14], high ability to cause a diverse array of life-threatening infections and its capacity to adapt to different environmental conditions [13,15]. The emergence of resistant strains is contributing nosocomial infections [16].

The emergence and the spread of multidrug-resistance *S. aureus* bacteria is a global threat for the therapeutic management of staphylococcal infections. Studies have shown that asymptomatic carrier children are a potential vector for the dissemination of MRSA in the community [17,18]. However, to our knowledge, the exact extent of MRSA in the children of Nepal has not yet been well assessed. Therefore, this study was an initiative to explore the distribution of MRSA in pediatric patients visiting International Children Friendship Hospital (IFCH). The presence of MRSA among a population of children were evaluated and assessed for the antimicrobial susceptibility profile. The infections in inpatients with those of outpatients were compared. The association between the causative organism with different variables such as age, gender and type of samples were assessed. These are important factors in *S. aureus*-related infections. We believe that this kind of study will aid physicians in administering first-line treatment by selecting the appropriate therapeutic drug against *S. aureus*.

## Materials & methods

### Materials

All the chemical, kits, antibiotic discs and microbiological media were purchased from HiMedia Pvt Ltd Co., Mumbai, India.

#### Sample collection

The study was conducted in the microbiology laboratory of IFCH, Kathmandu, during the period of June 2016 to November 2016. A total of 672 various clinical specimens viz. blood, urine, stool, pus swab from an ear, eye, throat, vaginal and body parts where burn or wound occurred were collected from both inpatients and outpatients. Only those patients who were not taking any medication and referred by a clinician were included in the study. Samples were stored in a sterile, leak-proof, screw-capped container with a proper label (Lab Id no., age, gender, date, time).

### Laboratory assessment

#### Isolation & identification

**Figure 1.**
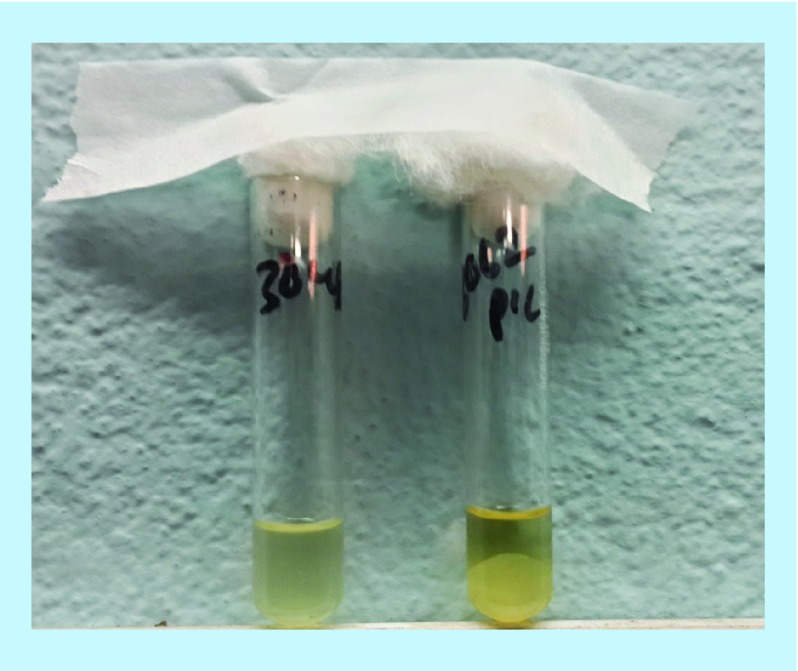
Photograph of tube coagulase test results (A) -ve and (B) +ve.

**Figure 2.**
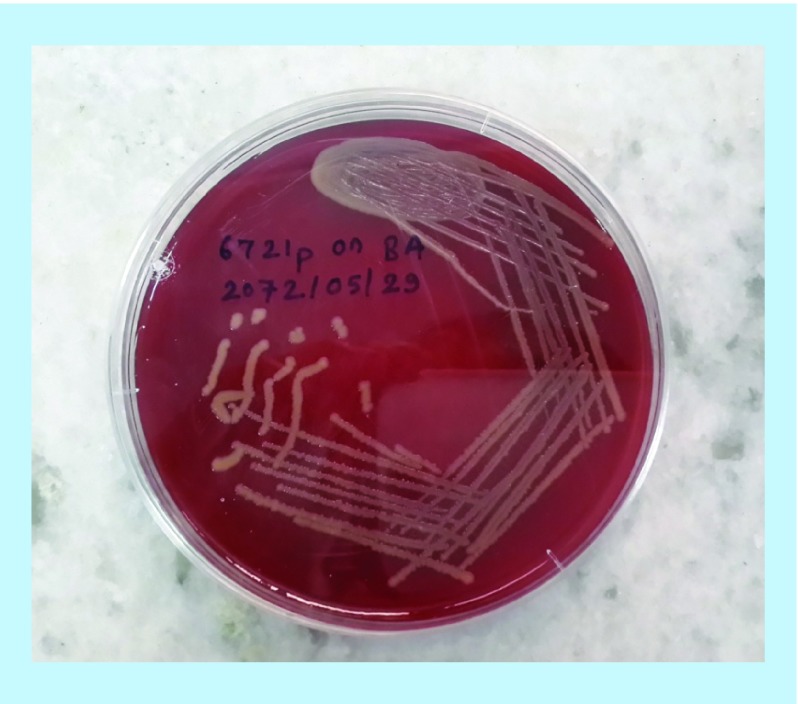
Photograph of *Staphylococcus aureus* in blood agar media.

The specimens were processed (i.e., gram staining for microscopic observation and microbial culture) in the microbiology laboratory within 2 h of the collection. A sample of blood was collected in the brain heart infusion (BHI) broth in 1:10 v/v ratio immediately, mixed well and then screw tightened the BHI bottle. The BHI bottles were then incubated at 37°C for up to 72 h followed by subculture on Blood Agar (BA) and MacConkey Agar (MA) plates. Samples such as pus, urine, stool, etc. were directly inoculated into BA and MA, whereas cerebrospinal fluid (CSF) samples were inoculated into MA and Chocolate (CA). Only the growth obtained in BA and CA plates were further cultured into Mannitol Salt Agar (MSA), an enrichment media for isolation of *S. aureus*. The BA and CA plates were incubated anaerobically (5–10% CO_2_), while MA and MSA plates were incubated aerobically at 37°C for 24 h. Colonies formed in MSA were picked and processed for gram staining. Only cocci were processed for the identification of *S. aureus*. For conformational identification of *S. aureus*, catalase test, coagulase (slide and tube) test were performed with the known positive and negative control strains (Figures 1 & 2).

#### Macroscopic examination

**Figure 3.**
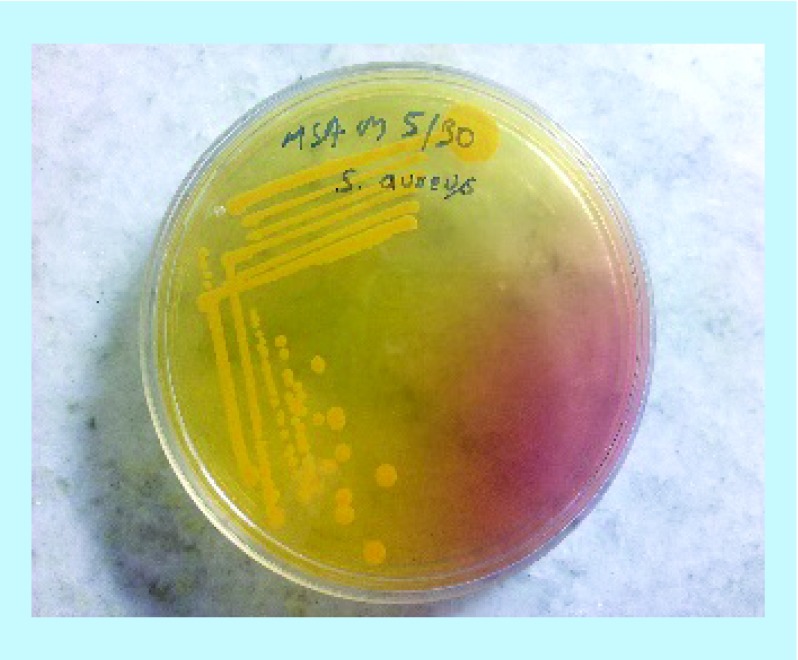
Photograph of *Staphylococcus aureus* in mannitol salt agar media.

Colony characteristics were examined on MSA and BA medium for the identification of all the isolates. β-hemolytic activity was detected on BA medium (Figure 3).

#### Antibiotic susceptibility

**Figure 4.**
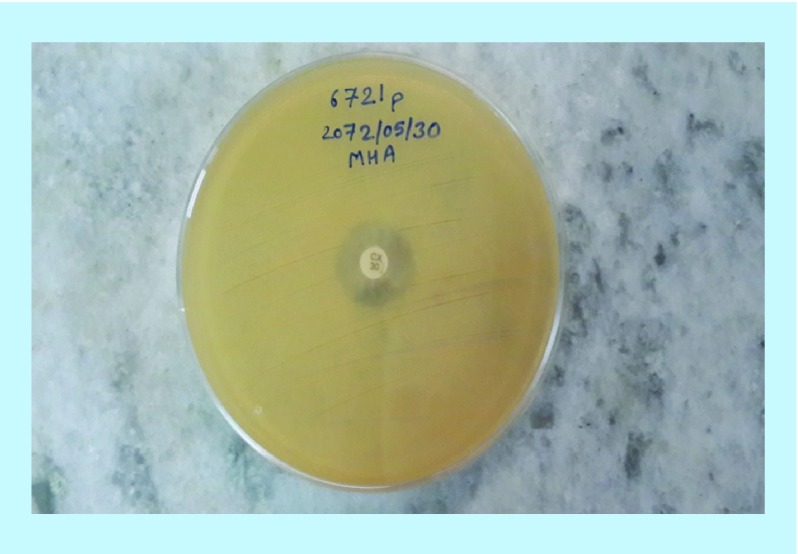
Photograph of *Staphylococcus aureus* in Mueller Hinton agar media showing methicillin resistance character against cefoxitin disc.

**Figure 5.**
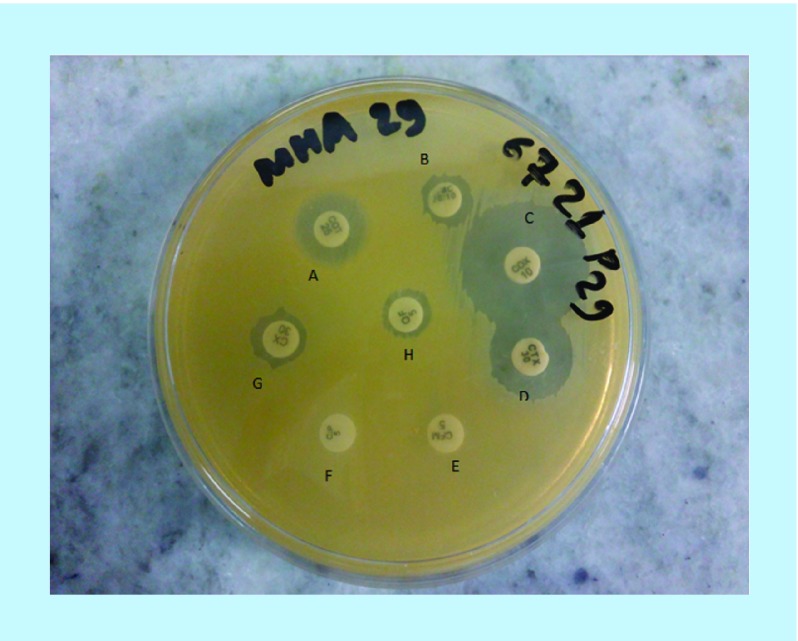
Photograph of antibiotic susceptibility test of methicillin-resistant Staphylococcus aureus in Mueller Hinton agar medium against various antibiotics. **(A)** Co-trimazaxole, **(B)** Chloramphenicol, **(C)** Cloxacillin, **(D)** Cefotaxime, **(E)** Cefixime, **(F)** Ciprofloxacin, **(G)** Cefoxitin and **(H)** Ofloxacin.

All identified *S. aureus* isolates from various clinical specimens were subjected to *in vitro* antibiotic susceptibility test by the Kirby–Bauer disc diffusion method following Clinical and Laboratory Standards Institutes (CLSI) guidelines [19]. The following 13 antibiotics at the indicated concentrations were tested against all the strains: cefoxitin (30 μ), penicillin (10 μ), ofloxacin (5 μ), erythromycin (15 μ), amoxicillin/clavulanic acid (15 μ), ciprofloxacin (5 μ), cotrimoxazole (25 μ), ampicillin (10 μ), amoxicillin (10 μ), cloxacillin (5 μ), azithromycin (15 μ), tetracycline (30 μ), gentamicin (10 μ) and vancomycin (30 μ). For identification of MRSA, cefoxitin disc (30 μ) was used. A zone of inhibition less than 22 mm or any discernible growth within zone of inhibition by *S. aureus* against cefoxitin in Muller hinton agar (MHA) plate was indicative of methicillin resistance. *S. aureus* ATCC 25923 was used as a standard control strain. Methicillin resistance was tested for all the *S. aureus* isolates by the agar screening method using MHA [20] supplemented with 4% NaCl against cefoxitin disc (Figures 4 & 5). For ICR identification, clindamycin and erythromycin drugs were kept apart 15–26 mm in MHA and D-shaped inhibition zone was observed around clindamycin.

### Statistical analysis

Microsoft Excel 2013 was used to record the laboratory data and statistical analysis software (IMB-SPSS V21.0) was used to calculate a p-value by using Pearson Chi-Square test. p > 0.05 was considered statistically significant at 95% CI.

## Results

### Isolation of *S. aureus*

Out of 672 clinical samples processed, bacterial growth was detected in 300 (44.64%) samples only. Among all bacterial isolates, *S. aureus* were isolated in 52 (17.3%) samples, comprising 29 (55.7%) isolates from inpatients and 23 (44.3%) from outpatients (Table 1).

**Table 1.** Distribution of *Staphylococcus aureus* in different age groups and types of patients.

Age group	Age ranges	Types of patients					
		Outpatients		Inpatients		Total	
		No.	%	No.	%	No.	%
Neonate	Newborn up to first 28 days	3	5.8	4.0	7.7	7	13.5
Infant	28 days–below 1 year	4	7.7	5.0	9.6	9	17.3
Toddlers	1 year–below 3 years	7	13.5	10.0	19.2	17	32.7
Preschool	3 years–below 5 years	6	11.5	4.0	7.7	10	19.2
School	6 years–10 years	3	5.8	4.0	7.7	7	13.5
Adolescent	11 years–below 15 years	0	0.0	2.0	3.8	2	3.8
Total		23	44.3	29	55.7	52	100.0

### Demography of *S. aureus*

Out of 52 *S. aureus*-positive samples, 35 (67.3%) and 17 (32.7%) were isolated from males and females, respectively (Table 3). Distribution of *S. aureus* in the toddler age group was the most (17 [32.7%]) and it was the least in the adolescent age group (2 [3.8%]) for both genders (Table 1).

### Distribution of MRSA in outpatients & inpatients

In addition to MRSA, response to cefoxitin was also stratified as methicillin-intermediate *S. aureus* (MISA) and methicillin-sensitive *S. aureus* (MSSA). The cefoxitin disc diffusion test showed that out of 52 *S. aureus* isolates, 39 (75.0%), seven (13.5%) and six (11.5%) were identified as MRSA, MISA and MSSA, respectively. Moreover, we found that among these 21 (72.4%), five (17.2%) and three (10.3%) of MRSA, MISA and MSSA, respectively, were isolated from inpatients. The analysis further showed that the incidence of MRSA and MSSA isolations (78.3%) and (13.0%), respectively, were slightly higher in outpatient than in inpatient samples. However, the association between the MRSA occurrence and inpatients was found statistically insignificant (p = 0.637 at 95% CI). On the other hand, the incidence of MISA isolation was lower in inpatient samples (six) compared with outpatient samples (2 [8.7%]). These data clearly show that frequency of MRSA in inpatients is higher compared with outpatient (Table 2).

**Table 2.** Distribution of methicillin-resistant *Staphylococcus aureus* in outpatients and inpatients.

Methicillin susceptibility	Inpatients no. (%)	Outpatients no. (%)	Total no. (%)	p-value
MRSA	21 (72.4)	18 (78.3)	39 (75.0)	p < 0.05
MISA	5 (17.2)	2 (8.7)	7 (13.5)	
MSSA	3 (10.3)	3 (13.0)	6 (11.5)	
Total no. (%)	29 (100.0)	23 (100.0)	52 (100.0)	

MISA: Methicillin-intermediate *Staphylococcus aureus*; MRSA: Methicillin-resistant *S. aureus*; MSSA: Methicillin-sensitive *S. aureus*.

### Distribution of *S. aureus* in various infection groups

Out of 35 samples from male patients, 23 (44.2%) were from skin and soft tissue infection (SSTI) patients (skin swab), seven (13.5%) were throat and nasal swabs samples taken from respiratory tract-infected (RTI) patients such as trachetis, sinusitis, laryngitis, pharyngitis/tonsillitis, rhinitis, acute otitis media, etc., five (9.6%) were blood samples and none (0%) were urine samples. Likewise, out of 17 samples from female patients, 11 (21.2%) were from SSTI patients, three (5.77%) were from RTI patients, two (3.9%) were blood samples and one (1.9%) was a urine sample. These results demonstrated that in both male and female pediatric patients, *S. aureus* strains were more prevalent in SSTIs compared with the remaining other types of infections (Table 3).

**Table 3.** Gender-wise distribution of *Staphylococcus aureus* in various infection groups.

Infection group	Male no. (%)	Female no. (%)	Total no. (%) of *S. aureus*
SSTIs	23 (44.2)	11 (21.2)	34 (65.4)
RTIs	7 (13.5)	3 (5.8)	10 (19.2)
Blood	5 (9.6)	2 (3.9)	7 (13.5)
UTIs	0 (0.0)	1 (1.9)	1 (1.9)
Total	35 (67.3)	17 (32.7)	52 (100.0)

RTI: Respiratory tract-infected; SSTI: Skin and soft tissue infection; UTI: Urinary tract infection.

### Antibiotic resistance pattern of *S. aureus*

The antimicrobial resistance (AMR) patterns of MRSA and MSSA isolates against antimicrobial agents are summarized in Table 4. More than 25% of MRSA isolates were resistant to ampicillin, ciprofloxacin, cotrimoxazole, erythromycin, clindamycin, azithromycin and tetracycline. Only a few of the MRSA isolates were found to be resistant to vancomycin (0%), cloxacillin (17.9%), gentamycin (20.68%) amikacin (10.2%) and chloramphenicol (23.1%). β-lactam antibiotics like cefoxitin (100%) and penicillin (100%) were found to be ineffective against MSSA too. While MSSA isolates showed resistance against ampicillin (69.2%) and ciprofloxacin (84.2%), the rest of the antibiotic showed less than 50% resistance toward the isolated MSSA.

**Table 4.** Antibiogram of methicillin-resistant *Staphylococcus aureus* and methicillin-sensitive *Staphylococcus aureus*.

Antibiotics used (μg)	MSSA (n = 13)		MRSA (n = 39)		Total sensitive N (%)	Total resistant N (%)
	S [n (%)]	R [n (%)]	S [n (%)]	R [n (%)]		
Cefoxitin (CX 30)	13 (100.0)	0 (0.0)	0 (0.0)	39 (100.0)	13 (25.0)	39 (75.0)
Penicillin (P 10)	0 (0.0)	13 (100.0)	0 (0.0)	39 (100.0)	13 (25.0)	39 (75.0)
Vancomycin (VA 5)	13 (100.0)	0 (0.0)	39 (100.0)	0 (0.0)	52 (100.0)	0 (0.0)
Cloxacillin (COX 5)	10 (76.9)	3 (23.1)	32 (82.1)	7 (17.9)	42 (80.8)	9 (19.2)
Ampicillin (AMP 10)	4 (30.8)	9 (69.2)	14 (35.9)	25 (64.1)	18 (34.6)	34 (65.4)
Ciprofloxacin (CIP 5)	2 (15.4)	11 (84.6)	18 (46.2)	21 (53.9)	20 (38.5)	32 (61.5)
Co-trimoxazole (COT 1.25/23.75)	9 (69.2)	4 (30.8)	22 (56.4)	17 (43.6)	31 (59.6)	21 (40.04)
Erythromycin (E 15)	7 (53.9)	6 (46.2)	21 (53.9)	18 (46.2)	28 (53.8)	24 (46.2)
Clindamycin (CD 2)	10 (76.9)	3 (23.1)	29 (74.1)	10 (25.6)	39 (75.0)	13 (25.0)
Gentamycin (GEN 10)	13 (100.0)	0 (0.0)	35 (89.9)	4 (10.2)	48 (92.3)	4 (7.7)
Chloramphenicol (C 30)	11 (84.6)	3 (23.1)	30 (76.9)	9 (23.1)	41 (78.8)	12 (21.2)
Azithromycin (AZM 15)	8 (61.5)	5 (38.5)	10 (25.6)	29 (74.4)	18 (34.6)	34 (65.4)
Tetracycline (TE 30)	12 (92.3)	0 (0.0)	19 (48.7)	20 (51.3)	31 (59.6)	20 (40.4)

MRSA: Methicillin-resistant *Staphylococcus aureus*; MSSA: Methicillin-sensitive *S. aureus*; R: Resistant; S: Sensitive.

### Susceptibility pattern of *S. aureus* toward macrolide-lincosamide-streptogramin B antibiotics

**Figure 6.**
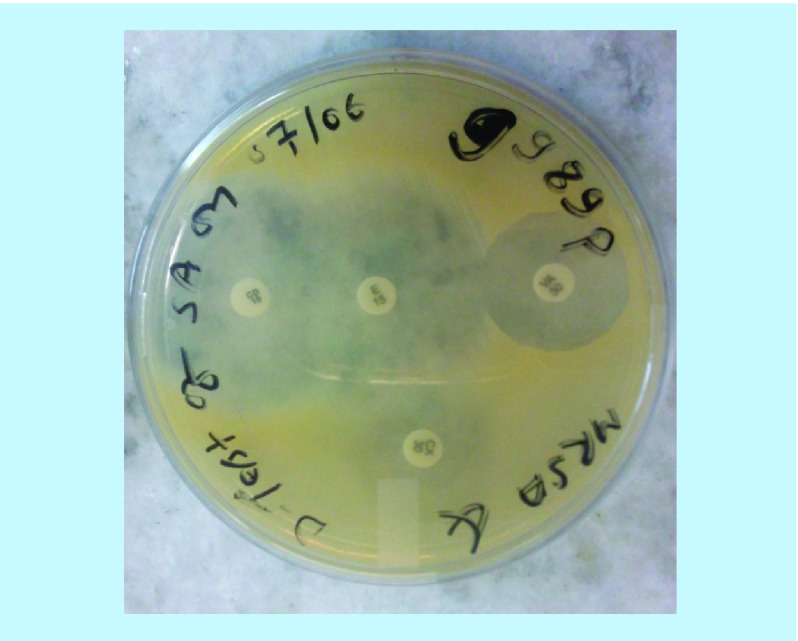
Inducible clindamycin resistant (D-test) positive showing MRSA in MHA media. CD: Clindamycin; E: Erythromycin; MHA: Muller hinton agar; MRSA: Methicillin-resistant *Staphylococcus aureus*.

Among 52 *S. aureus* isolates, inducible macrolide-lincosamide-streptogramin B (MLS_B_) resistance, constitutive MLS_B_, MS_B_ and susceptibility was found in eight (15.4 %), seven (13.5%), 17 (32.7%) and 20 (38.5%), respectively. Of the 39 MRSA isolates, 15.4% (n = 6/39) had inducible MLS_B_ resistance. Constitutive MLS_B_ was observed in 12.8% (n = 5/39) of MRSA isolates. In 38.5 % (n = 15/39) of isolates, MS_B_ resistance was observed and in 33.3% (n = 13/39) susceptibility to both erythromycin and clindamycin were observed. Out of 13 MSSA isolates, 38.5% (n = 5/13) were susceptible to both erythromycin and clindamycin, two (15.4 %) isolates each had inducible and constitutive MLS_B_ resistance and four (30.8%) isolates had MS_B_ resistance. Most of the MSSA were sensitive to both erythromycin and clindamycin antibiotics. This demonstrated that constitutive and inducible resistance was found higher among MRSA isolates as compared with MSSA (Table 5). The ICR or D-test-positive *S. aureus* is shown in Figure 6.

**Table 5.** Susceptibility pattern toward erythromycin and clindamycin.

Resistant and susceptible phenotype	Erythromycin	Clindamycin	D-test	*S. aureus* no. (%)	MRSA no. (%)	MSSA no. (%)
iMLS_B_	R	S	D+	8 (15.4)	6 (15.4)	2 (15.4)
cMLS_B_	R	R	–	7 (13.5)	5 (12.8)	2 (15.4)
MS_B_	R	S	D-	17 (32.7)	15 (38.5)	4 (30.8)
Susceptible	S	S	–	20 (38.5)	13 (33.3)	5 (38.5)
Total no. (%)	NA	NA	NA	52 (100)	39 (100)	13 (100)

ICR: Inducible clindamycin-resistant; MLS_B_: Macrolide-lincosamide-streptogramin B; cMLS_B_: Constitutive MLS_B_ phenotype; iMLS_B_: Inducible iMLS_B_ phenotype or ICR phenotypes; MS_B_: MS_B_ phenotype; NA: Not applicable.

### Grading of MRSA

Twenty-eight (71.8%) MRSA isolates were found to be multidrug-resistant oxacillin-resistant *S. aureus*, that is, MDR strains exhibiting resistant to ≥3 non-β-lactam antibiotics, and 11 (28.2%) MRSA isolates were found to be only oxacillin-resistant *S. aureus* that were resistant to ≤2 non β-lactam antibiotics. Association of multi-resistance observed with methicillin resistance is significant with (p = 0.03) at 95% CI (Table 6).

**Table 6.** Multidrug resistance pattern of methicillin-resistant *Staphylococcus aureus*.

Multidrug-resistant isolates			Total
MRSA	MORSA	ORSA	
Count	28	11	39
%	71.8%	28.2%	100.0%

MRSA: Methicillin-resistant *Staphylococcus aureus*; MORSA: Multidrug-resistant oxacillin-resistant *Staphylococcus aureus*; ORSA: Oxacillin-resistant *Staphylococcus aureus*.

## Discussion

*S. aureus* infection causes a health burden, particularly in low- and middle-income countries. MRSA is emerging as a serious threat to public health worldwide. MRSA has significantly increased morbidity and mortality rate of patients along with the increased length of hospital stay. *S. aureus* infections are a significant clinical problem in medical practice as the organism shows resistance to the commonly used first-line drugs. The prevalence of MRSA, however, varies markedly by country. In this study, the occurrence of *S. aureus* was studied among the pediatric patients visiting IFCH, using various types of sample. All the samples with clinically detected *S. aureus* may serve as a reservoir of MRSA, which may transmit the infection in a community. Thus, there is a chance of a rapid increase in the development of community-acquired MRSA infection. Though infected persons with *S. aureus* are a direct vital source, it is also reported to be isolated from fomites [21]. Fomites can be considered as an indirect transmission route for *S. aureus* as well as other infectious diseases in hospital [22]. So, environmental sources and fomites cannot be ignored.

In the present study, 52 (17.3%) *S. aureus* isolates were found out of 300 culture-positive samples. The results are similar to those reported by Mukhiya *et al.*, with growth positivity of 17.4% [23]. Here, the result also shows that children are more prone to *S. aureus*-associated infection. This might be due to high chances of contact with an infectious agent or their low immunity. Our findings show that *S. aureus* is a major cause of pyogenic infections. The skin and soft tissue (SST) was the most common site for the infection, likely because this bacterium normally inhabits the skin. Pandey *et al.* reported the higher isolation of *S. aureus* from pus and wound (78.37%) in comparison to blood (17.11%) and urine (4.50%) out of 111 *S. aureus*-containing clinical samples [24]. The current study also shows a parallel result, where 34 (65.4%) *S. aureus* isolates were found in SST samples, which are more than those obtained from RTIs, blood and UTIs.

In our study, when comparing the number and percentages of *S. aureus* in the Inpatient Department and Outpatient Department, the rate of *S. aureus* was higher in inpatients (55.7%) compared with outpatients (44.3%). Our result is in accordance with Rajbhandari *et al*. in Nepal [25]. They also reported the higher prevalence of *S. aureus* among the inpatient setting, accounting for 86 (62.3%) as compared with 52 (37.7%) in outpatients. Likewise, if we look at the distribution of MRSA isolates in different types of our patients, we found them to be 21 (40.4%) inpatients and 18 (30.6%) outpatients out of a total 39. These findings also indicate the higher prevalence of nosocomial infections. This higher occurrence of MRSA among inpatients could be due to various hospital associated risk factors such as prolonged hospital stay, antibiotic treatment, underlying immune-compromised condition, hospital environment, instrumentation and use of other invasive devices, which predispose patients to MRSA acquisition.

Over the course of time, *S. aureus* has developed resistance to different, conventionally used antibiotics. All the MRSA isolates were resistant to more antibiotics than MSSA isolates. A significant difference (p = 0.866) was observed in the case of cefoxitin, gentamicin, amikacin, cotrimoxazole and erythromycin. However, the difference observed in a case of tetracycline and ciprofloxacin was statistically insignificant (p > 0.05). Significant MDR cases of MRSA were 28 (71.8%). It also showed high resistance to cotrimoxazole and erythromycin, of 56.4 and 53.9%, respectively. These two antibiotics and mupirocin are usually used at random to cure a generalized and pyogenic infection. On the other hand, only 15.4% MRSA isolates showed ICR. The increasing rate of the Staphylococcal infections among the patients and the changing patterns of AMR has led to a renewed interest in the use of the clindamycin therapy in treating such infections [26]. The overall findings suggest an alarming situation of AMR accompanied by *S. aureus*. The development of antibiotic-resistant properties is troublesome and has been described as a serious public health concern, particularly in developing countries [27].

The study showed the frequency of MRSA to be 39 (75%). Another study carried out in Kathmandu valley by Shrestha *et al.* reported 44.9% of MRSA from nosocomial *S. aureus* [28,29]. Sanjana *et al.* also reported 348 (39.6%) MRSA isolates at College of Medical Sciences-Teaching Hospital [30]. Meanwhile, Rijal *et al.* in Pokhara valley reported 75.5% MRSA isolate and in a similar report, 69.1% MRSA isolate was found by Tiwari *et al*. in Western parts of Nepal [31,32]. The authors have attributed this to the indiscriminate use of antibiotics and its accessibility.

Prompt detection of MRSA may control for severe epidemics and lessen disease effects by allowing application of appropriate and timely treatment measures. Currently, various molecular techniques are used but they are neither economical nor feasible at small scale [33,34]. Availability of new drugs and inadequate establishment of MRSA treatment brings about the use of antibiotic combinations. Manytime new treatments and therapy are not available against MRSA, regardless of location. For MDR *S. aureus* strains, a combination of different antibiotics seems the only option. Tetracycline/sulbactam/cefoperazone combination has shown better results than the old combination of ampicillin/sulbactam/cefoperazone antibiotic [22]. This is also supported by similar minimum inhibitory concentration (MIC) results of others group’s data [35]. Some variations that occur may be due to differences in infection control measures, antibiotics prophylaxis, empirical therapy, treatments used in each ward and the clonal and often epidemic nature of these microorganisms.

The high rate of MRSA isolation and resistance to penicillin, ampicillin, ciprofloxacin, erythromycin, cotrimoxazole, chloramphenicol etc. shows that these antibacterial agents would be unreliable. Vancomycin seems to be the only antimicrobial agent that showed 100% sensitivity against *S. aureus* in the study. Hence, vancomycin may be used as the drug of choice for treating MDR-MRSA infections. However, frequent monitoring of vancomycin sensitivity and routine testing should be carried out. Use of vancomycin should be limited to preserve its value. It should be administered only in those cases where there is a clear need. Although all isolates were found to be sensitive to vancomycin, the screening test and MIC determinations are recommended for early detection of impending resistance and monitoring the response to therapy. The regular surveillance of nosocomial infections including monitoring antibiogram of MRSA and MSSA and formulation of definite antibiotic policy may be helpful in reducing the incidence of MRSA infection. The study is, therefore, an opening to facilitate epidemiological studies.

However, apart from antibiotic combinations for the treatment of MRSA, new treatment options using other antibiotics are slowly emerging. Nevertheless, these have their own drawbacks such as drug supply, cost and resistance itself. Therefore, this is not successfully being enforced in practice. Furthermore, the use of new antibiotics offers different antibiotic resistance mechanisms and new resistance properties are likely to emerge. Thus, the use of affordable antibiotics in suitable combinations is the only promising alternative method for MRSA-like bacterial infections. However, developed nations are working on making effective MRSA treatment strategies, including novel drugs or vaccines, gene therapy, bacteriophage engineering and so on by utilizing the latest tools and techniques. The authors of the current study recommend detecting novel resistance mechanisms such as *mec*C or uncommon phenotypes such as borderline-resistant oxacillin resistance.

## Conclusion

The occurrence of MRSA was found to be 39 (75.0%) out of a total 52 *S. aureus* isolates. The highest numbers of MRSA isolates were found in the toddler age group and male patients. Methicillin susceptibility of *S. aureus* was found higher in inpatients than outpatients. MRSA was found to be less resistant to gentamicin, cloxacillin and clindamycin, so these antimicrobial agents can be also used like mupirocin for the treatment of SSTIs and decolonization of MRSA carriers, but the use of such drugs should be limited. Alternatively, drugs like trimethoprim-sulfamethoxazole, teicoplanin, linezolid, quinupristin/dalfopristin, rifamycin, vancomycin and so on, or with suitable combination drugs are prescribed for MRSA treatment.

Both cefoxitin disc diffusion and D-test are the easy, reliable and cost-effective methods for detection of methicillin and inducible MLS_B_ resistance, respectively. Cefoxitin test and ICR (D-test) should be made compulsory in all *S. aureus* isolates in addition to routine susceptibility test methods, because the routine susceptibility test cannot detect MRSA unless cefoxitin disc is used. Likewise, inducible MLS_B_ resistance is not detected unless erythromycin and clindamycin antibiotics are placed 15–26 mm apart. It should be noted that these are time-consuming and labor-intensive methods. The sensitivity and specificity of these methods are also low and less reliable compared with other detection methods like MIC [36] determination, molecular techniques and so on. Though inducible MLS_B_ resistance is not associated with MRSA and widespread use of MLS_B_ has led to an increasing resistance to MLS_B_ antibiotics, they are still used to treat infections associated with *S. aureus*. This is also because of the safety and easy availability of these drugs compare to newer classes of antibiotics.

The study shows the MRSA occurrence is prevalent in pediatric patients. This corroborates the findings of previous researchers as discussed. Nevertheless, the total number of *S. aureus* isolates and the 6 months of study duration are low, which might be a possible source of errors and prevent drawing definite conclusions from this research. Newer classes of drugs are found to be more effective than β-lactam drugs to treat *S. aureus* infection. Moreover, MRSA infection is still one of the most life-threatening infections in hospitals. Therefore, regular surveillance of MRSA should be carried out in all hospital settings. In addition, restriction of the indiscriminate use of such antibiotics may be an effective strategy to control AMR. We should discourage empirical therapy practices, instead considering microbiological test reports. This might be an effective strategy to tackle the AMR problem and improve the infection treatment plan.

## Limitations

We were not able to perform a molecular test for genotyping or Staphylococcal cassettee chromosome mec gene (SCCmec) typing and similarly, MIC test for MRSA confirmation was not done due to lack of resources and time-bound factors. Neither risk factor analysis of MRSA carrier nor longitudinal data of MRSA trend was generated due to the limitation of current study settings and expertise in our network. Moreover, the study was conducted in a single hospital, so the findings do not represent a broad population and nonpatients.

## Future perspective

This study highlights the importance of routine testing, reporting and monitoring of MRSA, like in other drug resistance cases. Health care workers (HCWs) should be more conscious about AMR and nosocomial infections. In underdeveloped countries like Nepal, confirmation of microbial disease diagnosis and MDR should be done by molecular techniques despite them being uneconomical at small scale. The significant increase in constant AMR surveillance and monitoring of hospital associated infections (HAIs) should be facilitated in order to understand the nature of particular bacterial infections. Empirical therapy including combination drug prescriptions should exist, particularly in developing countries. The study shows male pediatric patients have higher MRSA than female pediatric patients, thus further study should be done to examine this relationship. Generally, male children are actively engaged in outdoor activities and thus exposed to the external environment more than female children – this could be one of the reasons for the higher incidence of bacterial infection in males.

**Summary points**

- Inducible clindamycin resistance was found to be higher among methicillin-resistant *Staphylococcus aureus* (MRSA) compared with methicillin-sensitive *S. aureus*.
- MRSA is emerging as a serious threat to public health worldwide. It adds burden to a patient by prolonging hospital stay and increasing morbidity and mortality rate.
- Vancomycin seems to be the only antimicrobial agent that showed 100% sensitivity against *S. aureus* in the study. Hence, vancomycin may be used as the drug of choice for treating multidrug resistance-MRSA infections.
- Although all isolates were found sensitive to vancomycin, screening test and minimum inhibitory concentration determination are recommended for early detection of impending resistance and monitoring the response to therapy.
- Apart from the antibiotic combination for the treatment of MRSA, new treatment options using other antibiotics are slowly emerging. Currently, the only promising option for developing countries is the use of affordable antibiotics in suitable combinations.
